# Supplementary material for: Are carbon emissions trading and green financial instruments synergistic? -Comprehensive quantitative research based on content analysis
Source: PLoS One. 2024 Mar 7;19(3):e0298601. doi: 10.1371/journal.pone.0298601 (PMC10919601; doi:10.1371/journal.pone.0298601)
Supplement: S1 Data — (ZIP) [file pone.0298601.s001.zip › Data/地级市Z_F工作报告.zip!/║┌┴·╜¡/╞▀╠¿║╙╩╨/└┤╫╘╣1⁄2╓┌║┼í╛┬φ┐╦╩2╛▌═°í┐_2011.docx]

各位代表：

　　现在，我代表市人民政府，向大会作政府工作报告，请予审议，并请列席会议的同志提出意见。

　　一、“十一五”和2010年工作回顾

　　“十一五”时期是我市发展史上极不平凡的五年。在省委、省政府和市委的正确领导下，在市人大、市政协的监督支持下，深入贯彻落实科学发展观，全面推进实施“358”跳跃式发展规划，超额完成“十一五”规划和跳跃式发展三年翻番目标。

　　——过去的五年，经济总量快速扩张，综合实力显著提升。全市生产总值、规模以上工业增加值、全口径财政收入、财政一般预算收入分别是2005年的2.6倍、3.8倍、3.15倍、3.96倍，年均增长21.1%、30.7%、25.8%、31.7%。累计固定资产投资是“十五”时期的4.4倍，年均增长37%。主要经济指标增速位居全省前列。

　　——过去的五年，产业结构优化升级，经济转型步伐加快。循环经济提档升级。呈现出产业规模扩大、产品链条拉长、技术工艺提升三大特点。地方煤矿由446处减少到217处，产能增加到1250万吨，34处矿井达到国家行业二级标准。洗煤企业由70个减少到40个，产能增加到3700万吨，用水全部实现闭路循环。焦炉由25座减少到13座，产能增加到900万吨，副产品全部成为下游产业原料。型煤又添新品种，新建生物质型煤项目2个，产能140万吨。煤化工项目从无到有，焦炉煤气制甲醇产能达到28万吨，煤焦油加氢和苯加氢产能均达到10万吨，结束了我市有焦无化的历史，煤焦油加氢技术填补全国空白，成为全省煤化工产业的排头兵。电力产业做大做强，大唐七台河发电公司两台60万千瓦机组并网发电，一批煤气、低热值煤发电项目投产，新增装机容量138万千瓦，成为全国重要的电力生产基地。非煤产业加快发展。木制品、机械、食品药品加工产业初具规模。木制品加工企业发展到102家、产能150万件套，双叶牌实木家具被评为中国名牌产品和驰名商标。规模以上机械加工企业发展到3家，被确定为全省六大农机装备产业园区之一。食品加工能力突破24万吨，制药企业发展到2家。开发区建设实现突破。投资4亿多元完善提升基础设施，超前收储土地，项目吸引力明显增强，已有9户企业入园，成功晋升为省级开发区。农业经济稳步提升。粮食产量连续五年超过10亿斤，畜牧业连续五年两位数增长。绿特色农业发展加快，新增标识认证273个。农副产品加工企业发展壮大，省级以上龙头企业达到7户。

　　——过去的五年，改革开放不断深化，发展活力日益增强。重点改革扎实推进。城乡一体化改革全省领先，城乡户籍、低保补差等八项政策标准实现统一。规范公务员及事业单位津贴补贴，财政供养人员工资水平居于全省前列。调整区县行政区划，市区面积新增1880平方公里，由全省末位跃升到第8位。完成集体林权制度主体改革。市电业局晋升为省直属局。城市信用社完成改制，入股龙江银行。非公重点企业加快发展，纳税超千万元的达到39户，超亿元的4户。发展环境持续改善。新建9个产业园区，承载项目超过200个。市政务服务中心建成运行，精简行政审批和收费项目77项，减免企业收费7亿多元。各级政府兑现优惠政策6亿多元。招商引资到位资金350亿元，新上固定资产投资亿元以上项目47个。对上争取成效显著。成功争取到国家循环经济、资源型城市经济转型、公立医院改革试点市政策，争取到各类项目450个、资金52.3亿元，创争取项目数量、资金额度、政策支持历史新高。对外开放日益扩大。成功举办三届中国·七台河家具节并升格为省级展会。融入东北东部“12+1”市州经济合作圈，与韩国曾坪郡等城市建立友好关系。开通直达俄罗斯乌苏里斯克的国际客货运输线路。累计外贸进出口总额是“十五”时期的14.6倍。

　　——过去的五年，城市建设全面提速，城乡面貌深刻变化。以创建国家园林城市为目标，加快推进城乡绿化、基础设施、滨水景观、“三供四治”、节能减排、环境治理等重点工程，是城市建设投资最大、项目最多、变化最显著的时期。绿化水平全面提高。“三年大造林”新增林地面积12.2万亩。新增绿化面积743.3公顷，新建改造公园广场15处、滨水景观带4处、小游园39处，市民出门“百米见绿、千米进园”，建成区绿化覆盖率、绿地率、人均公园绿地面积均达到国家园林城市标准。基础设施日趋完善。新建扩建七依高速、七勃公路、石油大道、桃山水库二期、汪清水库等一批重大基础设施项目，总投资超过100亿元。改造新建城区道路51条，新增供水管线34公里、供热能力1100万平方米、管道煤气用户4万户、排水管线19公里。“两型”社会建设初见成效。取缔严重污染环境的煤化工企业2户，关闭小煤矿229处、焦炉12座，关停超标排污小造纸厂18家，治理超标排放工业锅炉188台，实施环境污染治理项目82个、污染减排项目37个，万元GDP能耗累计下降25.8%。完成垃圾处理场及中转站等项目建设，治理矸石山和裸露山体3座，恢复受损土地面积100万平方米。

　　——过去的五年，社会事业长足发展，人民生活全面改善。社会保障显著增强。城乡低保做到应保尽保，有入住要求的农村五保户和城镇“三无”人员全部集中供养，“五七工”、“家属工”全部纳入养老保险统筹范围，高危行业人员全员参加工伤保险。提高城乡低保补贴、重点优抚对象、遗属和城镇“三无”人员、农村医疗救助、城市低保户供热、社区工作人员补贴标准，提高城镇职工医保报销比例和最低工资标准。社会事业全面进步。组建技师学院。新建改造中小学校舍75所、40万平方米。建设三级科技企业孵化器，成立宝泰隆博士后科研工作站、省级实木家具检测重点实验室。新建改造乡镇村卫生院（所）181所，市医院和七煤总医院晋升为三级甲等医院，政府为群众人均出资18元提供18项公共卫生服务。七台河籍运动员2006年和2010年冬奥会夺得金牌4枚，占中国所获金牌的近60%。住房条件明显改善。完成沉陷区治理工程，惠及1.55万户居民。棚改工程新建住宅小区3个、85万平方米。改造农村泥草房7410户，市区农村消灭泥草房。建设廉租房3170套、经济适用住房470套。群众收入持续增加。城镇居民人均可支配收入、农民人均纯收入、城乡居民人均储蓄存款比2005年分别增长1.03倍、1.04倍、1.02倍，年均增幅达到15.3%、15.8%、15.1%。

　　各位代表，过去的五年，是我市经济发展速度最快、总量快速扩张的五年，是运行质量效益最好、实力显著增强的五年，是城乡面貌变化最大、环境明显改善的五年，是群众得到实惠最多、收入大幅提升的五年。取得的成绩，让我们倍感骄傲；赢得的尊重，令我们无比自豪，一个政通人和、奋发进取、充满活力的七台河正昂首阔步迈向新的五年。

　　2010年是全面完成“十一五”规划的最后一年。全市上下紧紧围绕全省“双一”分解目标，抢抓机遇，攻坚克难，经济社会继续快速健康发展，超额完成市九届人大六次会议确定的目标。一是经济发展再上新台阶。全市生产总值实现305.2亿元，增长25.1%。规模以上工业增加值170.1亿元，增长38.7%，增速全省第一。全社会固定资产投资194.3亿元，增长43.5%。全口径财政收入47.5亿元，增长23.4%；财政一般预算收入23.3亿元，增长39%。二是产业转型实现新突破。煤炭循环经济快速发展。国有矿山西部煤炭资源深部勘查预获储量2.5亿吨，向阳90万吨矿井项目完成前期，新建扩建高产高效矿井8处，新增产能200万吨。完成鹿山和坤达两个120万吨、桃选90万吨洗煤项目，完成乾丰和隆鹏两个98万吨焦炉项目一期、美华60万吨焦炉项目，完成彩虹2亿块、北方2.4亿块煤建材砖项目。开工建设隆鹏100万吨和恒煤40万吨生物质型煤、鸿基200万吨粉煤灰水泥粉磨站、巨达6万吨环己酮和5万吨己二酸等项目。非煤接续产业发展壮大。新建金海4万吨和恒阳1万吨木塑产品项目，鑫泰10万吨矿用产品、永新12万吨助剂添加剂、旺族太阳能光电等项目进展顺利，恒益1500套电器控制及自动化设备、恒东200万平方米金属保温外墙板、经纬瓦斯抽放管项目投产。成功引进哈尔滨银行。5家小额贷款公司挂牌运营。运通物流园区投入使用。三是新农村建设再创新佳绩。粮食总产达到14.4亿斤，创历史新高。搬迁改造城中村3个，新建农民新区5个，改造农村泥草房4820户。新建扩建乡镇寄宿制学校5所、标准化卫生院（所）73个、标准化综合文化站（室）15个，完成7个中小型水库除险加固任务，解决16个村屯、1.33万人的饮水安全问题，试点村建设全部达标。四是改革开放迈出新步伐。完成政府机构改革，精简比例达到19%。引进固定资产投资亿元以上项目7个，千万元以上项目69个。对上争取项目120个、资金10亿元。争取资源型城市经济转型资金1.62亿元，矿山地质环境治理项目专项资金3亿元、到位1亿元。七台河海关监管场所得到批准。外贸进出口总额实现6096.3万美元，增长38%。五是城乡建设呈现新面貌。绿化村屯161个、造林9500亩，绿化乡村道路68条、195.5亩，建成区绿化面积新增50万平方米，高标准建设人民公园、荷花园等5个公园广场，顺利通过国家园林城市省级初审。人才路、景丰路等18条城市道路竣工。新建以弘扬奥运精神为主题的运动雕塑，城市文化品位进一步提升。实施治理减排工程11个，松花江流域水污染防治规划项目全面完成，关闭焦炉4座，淘汰落后产能57万吨。六是和谐社会建设取得新进展。校安工程加固28所学校校舍、面积6万平方米。公立医院改革试点扎实推进，创造性实施“3215”惠民工程和“868”人民满意工程，得到国家卫生部肯定。七台河籍运动员温哥华冬奥会夺得3枚金牌，王濛成为世界短道速滑首位“三冠王”。有线电视传输实现数字化。农家书屋建设经验在全省推广。双拥工作全面加强，被评为全省双拥模范城。连续29年无森林火灾。深入开展严打整治活动，群众安全感普遍增强。积极做好信访工作，重点信访案件结案率达到92.5%。七是人民生活质量又有新提高。城镇人均可支配收入、农民人均纯收入分别实现15000元、6955元，增长9.5%、25%。开发公益性岗位安置困难群体就业1370人，城镇登记失业率低于省控指标1.2个百分点。提高城镇职工医疗保险年度统筹、大病统筹、城镇居民基本医疗保险和新型农村合作医疗最高支付限额，提高原国有关闭、破产、改制企业退休人员个人账户记入比例。新增住房租赁补贴家庭1000户，完成金厦佳园二期3万平方米廉租房建设。八是依法行政水平得到新提升。认真执行市人大及其常委会的决议、决定，人大代表议案、意见和建议办复率达到100%。“五五”普法通过国家验收。市政务服务中心触角延伸到基层，形成一个中心、五个分中心、22个街道社区服务中心的全方位服务网络。清理行政事业收费项目46项,优化发展环境做法得到省委肯定。

　　统计、审计、外侨、食品药品、计生、老龄、气象、地震、人防、地方志、档案、民兵、预备役等工作取得新成绩，民族宗教、红十字、妇女儿童、残疾人等事业长足发展。

　　各位代表，回首过去五年的发展历程，七台河在经历宏观环境新变化、经受复杂局面新考验中，发生的变化令人欣喜，实现的发展令人振奋，取得的成就令人瞩目。这些成绩的取得，是省委、省政府和市委正确领导的结果，是市人大、市政协和社会各界有效监督与鼎力支持的结果，是中省直单位无私奉献、全力配合的结果，是全市上下群策群力、拼搏实干的结果。在此，我代表市人民政府向各位代表和全市人民，向所有关心、支持七台河发展的各界人士，表示崇高的敬意和衷心的感谢！

　　在肯定成绩的同时，我们也清醒地看到存在的问题。主要是煤炭循环经济链条短、规模小、效益低，企业联合重组、产业优化升级任务艰巨；非煤产业基础薄弱、人才短缺、发展不快、比重过小，加速城市经济转型形势紧迫；城市基础设施承载力不强、生产服务功能不完善、生活服务产业不发达，加快城市化进程任重道远；重化工产业比重大、能耗高、污染重，资源环境约束日益加剧，实现低碳、绿色发展压力增大；社会管理机制有待进一步健全，民生保障有待进一步加强，公共服务有待进一步完善，行政效能有待进一步提高，煤城文化有待进一步升华等。

　　二、“十二五”时期的宏观环境和基本构想

　　未来五年，是七台河大有作为的战略机遇期。全球经济结构的深度调整、产业格局的深刻变化、科技创新的深入发展，为我们改造提升传统产业、引进发展新兴产业，创造了良好的外部环境。全国由初级工业化向后工业化过渡，城市化进程加速，重化工业新型化加快，为我们完善提高城市硬环境，打造提升城市软实力，做精做强煤炭循环经济，发展壮大非煤优势产业，创造了难得的发展机遇。全省“十二五”规划提出“两个翻番”的奋斗目标，区域竞争、城市比拼日趋激烈，为我们实现更好更高更快发展，创造了强劲的内生动力。全市经济增幅连续三年超过25%，煤、焦、电、化主导产业的形成，为我们开创建设龙江强市新局面，创造了坚实的产业基础。

　　基于这一历史发展背景，提交大会审议的《七台河市国民经济和社会发展“十二五”规划纲要（草案）》，提出了“全面加快强市步伐，努力实现更高跳跃”的奋斗目标，确立了更好更高更快发展的“818百亿工程”，即2015年全市生产总值实现800亿元,全口径财政收入实现100亿元，固定资产投资完成800亿元。突出强调坚持循环发展、多元发展、绿色发展、创新发展、统筹发展、共享发展六条基本原则，抓好调整优化空间布局、加快推进经济转型、建设生态园林城市、加强资源节约和环境保护、加快科教人才兴市步伐、促进公共服务均等化、深化改革扩大开放、加强社会主义民主政治和精神文明建设八项主要任务，并制定了规划实施保证措施。

　　各位代表，“全面加快强市步伐，努力实现更高跳跃”，是我们站在新的历史起点上，提出的更好更高更快发展目标。既充分考虑了广大人民群众盼发展、求富裕的强烈愿望，又统筹兼顾了我市现实的产业基础和未来的项目支撑，是经过反复研究论证，通过努力工作，完全可以实现的目标。到2015年，七台河将成为经济实力更加雄厚、社会事业更加和谐、城市环境更加优美、人民生活更加富裕的龙江强市，让我们为实现这一美好愿景而努力奋斗！

　　为确保《纲要》的顺利实施，必须努力做到“五个并重”。

　　一是城市化与工业化并重。城市化与工业化，是实现现代化的两条必由之路，城市化带来需求、工业化提供供给，两者相互依存、相互促进。目前，我市城市化明显滞后于工业化，不仅道路交通等基础设施难以承载工业化的快速推进，就医、住房等生活条件也不能满足工业化加速集聚人才的需要，特别是金融、资本、咨询等生产服务业不能适应企业和个人低成本、便捷式交易需求，严重阻碍工业化发展。加快推进城市化，促进城市化与工业化协调发展、相互助推，是“十二五”时期的重要任务。

　　二是转型产业与主导产业并重。实践证明，资源型城市经济转型需要一个较长的过程，七台河的城市经济转型同样不可能一蹴而就。从稳定经济社会整体运行的角度出发，我们必须坚持巩固提升煤炭主导优势产业，科学有序开采有限的煤炭资源，大力发展煤炭循环经济，向现代煤化工产业迈进，为城市经济转型赢得时间和创造条件。同时，坚持加快发展非煤转型产业，做大现有非煤优势产业，研发引进新材料、新能源等环保低碳项目，做到煤与非煤两手抓、两手硬，两不误、两促进。

　　三是发展经济与保护环境并重。我市主导产业多为重化工业，资源依赖强、能源消耗高、环境污染大。面对日趋强化的资源环境约束，我们必须牢固树立低碳、绿色发展理念，坚持经济发展与环境建设同步规划、同步实施、同步收效，实现经济效益与生态效益相统一、人与自然相和谐，着力建设资源节约型、环境友好型社会。

　　四是加快发展与共享发展并重。经济加快发展，是群众共享发展的物质基础，群众共享发展是经济加快发展的根本目的。我们必须一手抓发展不放松，创造更多物质财富，为改善提高民生创造条件、提供支持；一手抓民生不动摇，把改善民生作为一切工作的出发点和落脚点，着力解决就业、医疗、教育、社保、住房等民生问题，切实让人民群众在经济加快发展的同时分享发展成果。

　　五是谋划长远与立足当前并重。没有当前发展的逐步积累，就没有长远目标的最终实现。同样，只有长远目标的真正实现，才会有持久的“眼前利益”。我们既要立足当前，积极应对全球金融危机对经济发展的后续影响，抓住世界经济结构进入调整期特点，努力实现经济社会平稳较快发展。同时，又要深刻分析和准确把握经济发展的中长期趋势，科学制定发展规划和调整城市发展定位，把工作的着力点放在长远发展上，多做打基础、利长远、增后劲的事。

　　三、2011年总体要求和主要任务

　　2011年是实施“十二五”规划的开局之年。政府工作的基本思路和总体目标是：高举中国特色社会主义伟大旗帜，以邓小平理论和“三个代表”重要思想为指导，深入贯彻落实科学发展观，全力推进省委建设“八大经济区”、实施“十大工程”战略部署，以科学发展为主题，以转型发展为主线，以包容发展为模式，以安全发展为保证，以共享发展为目的，坚持工业化与城市化同步推进，主导产业与转型产业协调发展，更加注重生态建设，更加注重改善民生，更加注重社会和谐，全面开创建设龙江强市、实现更好更快发展的新局面。全市经济社会发展的主要预期目标是：地区生产总值增长22%，财政一般预算收入增长16%，全社会固定资产投资增长35%，城镇居民人均可支配收入增长15%，农民人均纯收入增长15%。

　　为实现上述目标，重点抓好以下七项工作。

　　（一）突出强化产业项目建设，着力加快转变发展方式。坚持把产业结构优化升级作为转变发展方式的主攻方向，把项目建设作为加快转型的第一抓手，以超前的眼光、务实的措施、细致的工作，确保建成投产一批投资亿元以上的大项目，坚决打胜产业项目建设攻坚战，通过上项目实现扩总量、优结构、促转型、增效益。

　　一是坚持巩固提升煤炭主导产业不动摇，全力发展循环经济。原煤开采业立足超前应对国家新一轮煤矿整顿关闭，加快资源勘探、新矿井建设、老矿井技改扩能、企业整合重组，完成国有矿山西部煤炭资源深部勘查项目和一批矿井改造扩能工程，开工建设向阳90万吨、亿达信60万吨矿井，推进宝泰隆种畜场60万吨矿井前期，力争矿井数量减少但产能不减，地方原煤产量稳定在700万吨。煤炭洗选业立足提升行业整体水平，通过严格的产业、环保政策，督促企业改进工艺，关闭浪费资源、污染环境、工艺落后的洗煤企业。型煤加工业立足培植骨干，完成隆鹏100万吨和恒煤40万吨生物质型煤项目，带动整体行业发展。新型建材业立足市场开拓，政府投资工程优先使用，民间投资工程达标使用，煤建材砖使用率提高10%。焦炭加工业立足规范提高，督促引导企业完善工艺、上齐设施，力争做到全部实现干法熄焦、热量综合利用，同时焦炉煤气等副产品全部回收转化升值，完成普能90万吨、隆鹏98万吨焦炉项目二期建设。煤化工产业立足深度开发，大力发展合成化工、材料化工、精细化工，启动博兴化工45万吨合成氨和80万吨尿素、宝泰隆公司60万吨煤焦油精深加工、七煤公司30万吨焦油深加工和10万吨炭黑项目，完成巨达6万吨环己酮和5万吨己二酸项目。加强与中国矿大合作，谋划6万吨针状焦、5万吨高速公路沥青和500吨高性能炭纤维等新材料项目。电力产业立足做大规模，继续推进低热值煤发电项目，大力发展风电、瓦斯煤层气发电项目。开工建设七煤公司30万千瓦矸石发电项目，全力推进七里嘎山4.95万千瓦风电项目。二是坚持发展壮大非煤接续产业不放松，全力推进转型发展。加快推进新兴木制品园区建设，引导企业入园配套发展，支持有条件的企业加速扩张。抢抓被列为全省六大农机装备制造园区政策机遇，规划建设勃农兴达农机装备产业园，发挥机械制造传统品牌和矿山机械销地优势，支持机械制造企业加快发展。完成霸州钢构、安泰2万件精密铸造项目，新建隆泰62万平方米无比钢抗震墙体构件、华丽20万平方米轻钢结构等转型项目。利用周边丰富的农副产品资源，突出发展食品药品加工大项目，完成嘉美5000吨大豆纤维、戈比格1万吨野生果酒项目，建设叶淼1500吨保健露项目，促进七台河制药厂与三精药业合作项目健康运行，进一步扩大合作范围。发挥本地体育名人效应，推进体育服装和器材产业发展。三是坚持引进发展新兴产业不懈怠，全力培育新的经济增长点。立足现有资源和产业基础，重点开发多孔陶瓷、抗菌陶瓷、新型保温材料等新材料产品，引进纳米汽车电池等高新技术项目，发展节煤增效剂、电机节电器、通用变频器等节能产品，推广煤矸石、粉煤灰、作物秸秆等综合利用项目，促进城镇污水、工业废水深度处理等环保产业。重点完成弘远40万平方米建筑节能EPS模块、永新12万吨助剂添加剂、东响超细玻璃纤维、旺族太阳能光电等项目，加快推进华纳无源器件项目，积极引进中科院汽车电池等新兴产业项目。

　　（二）突出抓好招商引资，着力增强经济发展后劲。坚持把招商引资作为促进项目建设、推动经济发展的长效举措，加强力量、搭建平台、优化环境、严格奖惩，全面提升招商引资质量效果，力争引进固定资产投资10亿元以上项目2个，亿元以上项目10个。

　　一是全面强化招商引资措施。强化规划招商，突出规划的引领作用，以高质量、操作性强的规划，引导域内企业扩大投资，增强域外客商投资信心。强化产业招商，依托现有优势产业，引进换代升级和上下游项目。强化定向招商，瞄准重点区域、重点企业、重点项目走出去招商。强化以商招商，以良好的亲商安商富商效果，吸引投资者主动招商。强化以企招商，引导企业增强开放意识，与国内外知名企业合作发展。强化专业招商，发挥专业招商组示范作用，引进规模大、质量高、前景好的重大项目。强化项目招商，研发储备一批经济效益高、吸引能力强的项目对外招商。强化责任招商，明确招商引资任务，严格招商引资考核，督促政府公务人员招商。强化招商引智，加强与大专院校、科研院所合作，积极引进发展博士经济、硕士产业。二是创造更加优良的发展环境。进一步调优招商引资政策，及时兑现招商引资和扶持企业发展承诺。设立产业发展专项资金，发挥好中小企业、木制品产业、科技项目专项资金作用，协调金融部门扩大企业贷款规模，支持具备条件的企业上市融资、发行债券，有效解决资金问题。抢抓新一轮土地利用总体规划修编机遇，超前预留项目用地，着力解决征地难题。完善政务服务中心运行机制，实行项目审批全程代办制，切实提高行政效能。三是加速推进经济开发区建设。围绕打造转型产业承载区、城市功能新区，高标准完成开发区整体功能规划，超前谋划建设基础设施，全面完善提升功能，切实增强项目承载力。加强与哈尔滨理工大学合作，加快建设国家高科技创业园分区，积极筹建省级新材料产业园区。全面落实开发区优惠政策，吸引更多符合条件的项目入园，力争引进项目10个以上。四是扎实搞好对上争取。深入研究国家宏观政策，准确掌握投资方向和重点，围绕城市经济转型、农田水利设施、小城镇建设、重点支持产业、大型农机合作社等高标准谋划一批重大项目，加强与国家和省相关部门的沟通联系，力争域外煤炭资源划拨、跨区域调水等重大项目实现突破，对上争取资金不少于10亿元。

　　（三）突出加强“三农”工作，着力推进城乡一体化。坚持农民增收、农业增效、农村繁荣目标不动摇，全面推进农民市民化、农业现代化、农村城镇化，加速城乡一体化。

　　一是大力发展现代特色农业。全力提升集约化、机械化、标准化、科技化种植水平，积极推广优良品种，继续扩大绿特色作物面积，粮食总产稳定在14亿斤以上。加快建设集中连片蔬菜基地，新增棚室蔬菜面积1000亩以上。加强农田水利基础设施建设，超前做好防汛、抗旱工作。畜牧扶持政策重点向主导养殖品种、养殖大户、标准化小区、专业村倾斜，完善良种繁育、疫病防治和畜牧生产安全预警体系。二是全面提升农村经济市场化程度。引进扶持农畜产品加工项目，引导发展紧密型的“企业+基地+农户”产业化经营模式，培育壮大水稻、大豆、玉米、蔬菜、肉鸡、生猪、大鹅7条农畜产品加工产业链，带动形成种植基地150万亩以上，促进生猪、肉鸡等主导品种存出栏实现两位数增长。鼓励农户加快建立以资金、技术、劳务和农产品营销等为纽带的农村合作组织，引导发展多种形式的土地适度规模经营，新增土地流转面积30万亩以上。三是加快推进小城镇建设。综合运用城中村改造、农村泥草房改造、危房重建、自然屯撤并政策，加快推进万宝河、双河2个省级百镇建设工程试点，启动中心河、种畜场第六作业区2个市级小城镇建设试点，完成茄子河区鹿山村等5个自然屯异地新建，改造万宝村等5个城中村，搞好农民新区、农民公寓建设。四是整体推进新农村建设。着力构建政府主导、农民主体、企业帮建、部门包扶的投融资体系，完成100个中心村规划，搞好20个示范村、27个重点村试点建设。积极做好农村饮水安全、新能源、道路、教育、医疗、社会保障等民生工作，改善农村生产生活条件。建设学习型村屯，培育新型农民。

　　（四）突出完善城市功能，着力加速城市化进程。高标准完成城市总体规划修编，全面加快旧城改造，着力提升城市功能，打造适宜人居、利于创业的山水园林城市，大幅增强城市综合竞争力。

　　一是调整优化产业布局，完善提高城市生产功能。坚持因地制宜，充分发挥区域比较优势，科学调整区、县、场生产功能定位。统筹考虑产业空间布局，合理确定园区功能，规范现有产业园区，加快建设现代产业集中区，形成布局优化、要素集聚、项目配套、产业集群、特色鲜明的园区体系。二是创建国家园林城市，完善提高城市生态功能。新建茄子河、新兴、勃利等公园，完善人民公园、应龙山植物园绿化，高标准完成学府街东延、石油大道、开发区大道两侧绿化带工程，全力搞好万宝湖坝下风景区、新老七台河、万宝河等滨水景观带绿化建设，加快桃山湖坝下风景区和房地产综合开发项目建设。扎实推进村屯、公路、小区、庭院、库渠堤坝绿化和荒山荒地造林，强化湿地保护和森林生态建设。全面推进“两型”社会建设，实施污染物排放总量控制，严格焦化企业环保监管，强化节能降耗监测监控，加强水源地生态保护，突出市容市貌、大气污染、矸石山、裸露山体、厂矿环境、居民小区、棚改搬迁腾空土地治理，全面改善矿山生态环境，确保实现国家园林城市创建目标。三是加快基础设施建设，完善提高城市承载功能。超前谋划建设城市基础设施，实施铁路新客运站和站前广场工程，加快七密铁路项目前期，争取勃利至林口铁路复线、七台河至鸡西城际铁路项目。启动公路客运总站项目，完成七依高速公路主体工程，石油大道、七勃公路实现全线通车，积极谋划七密和七宝高速公路、南环绕城运煤通道、七桦公路市区至西长发收费站高速改扩建工程。推进桃山水库二期项目，完成汪清水库增容扩建工程和九龙水库建设。加快建设庆云500KV、勃利220KV、北兴220KV输变电工程。搞好大同街、大明街等城区道路改造和倭肯河两岸治理，全力推进“三供四治”工程。四是加快发展商务服务产业，完善提高城市生产服务功能。加快引进兴业银行，支持哈尔滨银行网点运营，促进小额贷款公司健康发展，推进宝泰隆公司早日上市，加快发展资本市场。搞好新兴、桃山、华鑫物流园区建设，大力发展第三方物流。发挥体育会展中心载体作用，承办高规格体育赛事，办好第四届七台河家具节。积极发展电子商务、服务外包、信息等现代服务业，大力引进咨询、评估、工程设计、审批代办等商务服务业，着力打造最便捷的交易平台，降低交易成本。五是改造传统服务产业，完善提高城市生活服务功能。编制北国风光特色旅游开发规划，加快旅游名村名镇建设，大力发展边境游和跨境游。有序发展房地产业，加快保障性住房建设，提高普通商品住房供给比例，适度增加功能性住宅。加大公交投入，改善公交设备设施，整治交通环境，规范交通秩序，提高服务质量。大力发展文化娱乐、体育健身项目，谋划建设文化中心，优化发展餐饮业，突出发展社区服务业，全力实施名医、名校工程，全面改善生活环境。

　　（五）突出深化改革扩大开放，着力提升经济社会发展驱动力。坚持把改革开放作为破解发展难题、实现跳跃前进的强大动力，切实解放思想，强化措施，不断增强经济社会发展活力。

　　一是积极稳妥地推进重点领域改革。深化医药卫生体制改革，加快推进国家公立医院改革试点，全面实施基本药物制度，促进基本公共卫生服务均等化。完成政府机构改革收尾工作，理顺开发区、种畜场管理体制。改革城市建设投融资机制，扩大融资平台规模，力争发行市政债。深化农村综合改革，全面完成集体林权制度改革，完善“三资”委托代理服务制。二是全力促进非公经济加快发展。积极支持全民创业，不断强化政策扶持，努力轻费减负，降低创业门槛，深入开展创办小企业、开发新岗位和培育“万名小老板”活动。全面落实国务院鼓励民间投资的“新36条”，实行“非禁即入”，打破行业垄断，坚持外商内商一视同仁，外资内资一样优惠，鼓励本地企业和民间资本投资兴业，确保非公企业固定资产投资增长50%以上。大力实施中小企业成长工程，力争15户中小企业进入规模以上行列，新增纳税超亿元非公企业1户、超千万元非公企业2户。三是全面扩大对外开放。培育壮大外贸进出口企业群体，扶强扶优骨干出口企业，着力打造出口品牌，完成海关监管场所建设，启动荣盛达国际物流中心，外贸进出口总额实现7015万美元，增长15%。实施“走出去”战略，鼓励企业扩大境外投资，开展跨国经营，大力发展境外劳务经济。

　　（六）突出加强社会事业建设，着力保障和改善民生。坚持把加快发展社会事业作为改善和保障民生的首要措施，全面加强社会事业建设，具体实施七项民生工程、办好20件实事，让人民群众共享改革发展成果。

　　一是实施安居建设工程。加快推进保障性住房建设，抓好农村泥草房、危房改造和廉租房、公共租赁房、经济适用房和商品房建设，满足不同群众住房需求。加强房地产市场监管，遏制房价过快上涨。（1）打好棚户区改造攻坚战，力争两年完成建设任务。今年完成3个棚改小区续建工程，搬迁安置居民1.5万户；新建5个棚改小区、170万平方米。（2）新建廉租住房2124套、公共租赁住房1456套、经济适用住房200套，确保土地出让金净收益的10%用于廉租房建设。住房租赁补贴标准由每月60-80元提高到90-150元，新增保障补贴家庭2500户。（3）改造农村泥草房7254户。二是实施保障惠民工程。严格落实国家和省社会保障政策，不断加大财政投入，全面增强社会保障能力。（4）失业保险实行市级统筹，适当提高失业金支付标准。（5）按照国家、省有关政策，妥善解决原国有企业、集体企业、改制企业老工伤人员和工亡职工供养亲属待遇问题；解决农林牧渔四场、刑满释放劳教人员等参加养老保险问题；解决城镇老年居民基本养老保障问题。（6）企业离退休人员工资上调10%，企业用工最低工资标准提高10%以上。（7）逐步扩大参保患者门诊统筹实施范围，推行市内定点三级、二级医院医保资金预付制和住院医保患者即结即报制，城乡参保居民住院报销比例达到60%以上。（8）城乡居民医保财政补助水平由120元提高到200元，人均基本公共卫生服务经费由18元提高到25元以上。（9）城乡低保补助标准提高28元，财政补助水平月人均30元。（10）提高困难学生资助标准，特困生资助实现全覆盖。（11）提高儿童福利机构供养及社会散居孤儿生活补助标准。（12）免费实施白内障复明手术100例，为残疾人提供用品用具及辅助器具300件。三是实施社会进步工程。（13）按照优先发展、育人为本、促进公平、改革创新、提高质量的总体要求，加强学前教育，加快公办幼儿园建设；推进标准化学校建设，促进义务教育均衡发展；实施名校、名师工程，提高高中教学质量；强化职业教育基础能力建设，加快职业教育发展。（14）加快推进国家公立医院改革试点，建设市医院外科楼、妇幼保健院、中医院、精神病医院和4个社区卫生服务中心、62个乡镇村卫生院（所），引导优质卫生资源向基层转移，着力构建覆盖城乡的便民医疗卫生服务网络，有效解决看病难、看病贵问题。（15）全面实施计生“惠家工程”，为4.5万名已婚育龄妇女进行生殖健康免费检查，城市独生子女父母奖励费实现当年及时兑现，陈欠逐年还清。（16）打造10个城市示范社区和10个农村样板社区。四是实施就业富民工程。（17）把促进充分就业作为经济社会发展的优先目标，实施更加积极的就业政策，全面落实全民创业扶持政策，实施高校毕业生就业援助彩虹工程、助残就业爱心工程，加大零就业家庭援助力度，开发公益性岗位500个，城镇登记失业率低于4.3%。五是实施饮水安全工程。（18）强化水源地环境综合治理，实施水库上游农村环境综合整治示范工程，完成富铁小区及茄子河镇污水截流工程，确保污水处理厂正常运营。六是实施稳价保供工程。（19）严格落实中央和省有关政策，降低流通成本，兑现各种价格补贴，用好用活价格调节基金，适时平抑物价。依法做好物价监管，严厉打击违法涨价行为。七是实施便民出行工程。（20）加大财政支持力度，优先发展城市公交，优化交通网络布局，增加客运班次，方便居民出行。依法规范客运市场秩序，坚决打击出租车随意涨价行为。

　　各位代表，本届政府坚持以改善提高民生为根本宗旨，倾注更多的感情和精力，组织更多的物力和财力，全面改善城乡居民生产生活条件，今年民生投入将超过20亿元，力求让七台河每个市民生活得更加富足、更加健康、更加舒心、更有尊严。

　　（七）突出做好安全生产工作，着力维护社会和谐稳定。牢固树立抓安全就是抓发展、保稳定就是保发展理念，全面强化安全稳定措施，保障经济社会更好更快发展。

　　一是强化安全生产工作。全面落实安全生产责任制，加强井下避险“六大系统”建设，严格日常安全监管，强化隐患排查治理，确保煤矿不发生较大以上事故。突出加强食品药品安全监管，建设食品药品检验检测中心。全面抓好消防、交通、易燃易爆和危险化学品、非煤矿山、森林防火、特种设备等领域的安全工作。二是强化公共安全管理。健全安全预警信息系统和应急联动机制，完善突发事件应急预案，开展突发事件应急演练，提高保障公共安全、处置突发公共事件、防灾减灾和维护社会稳定能力。三是强化社会治安综合治理。扎实推进“平安七台河”建设，完善社会治安防控体系，推进传统治安管理向社会管理转变，始终保持对违法犯罪的高压态势，有效遏制恶性案件发生。四是强化信访稳定工作。严格落实信访责任制，完善矛盾预警和排查调处机制，推广三级联动视频接访和信访代理，畅通群众诉求渠道，下大力气解决群众反映强烈的突出问题，维护社会公平正义。

　　四、全面加强政府自身建设

　　坚持以建设人民满意服务型政府为目标，全力打造职能有限、行政有为的政府，民主法治、执行力强的政府，清正廉洁、取信于民的政府。

　　（一）坚持科学理政。坚持科学、民主、依法决策，严格执行重大事项集体决策、专家咨询、公示听证、决策评估制度，提高公共政策制定的公众参与度。科学履行经济调节、市场监管、社会管理、公共服务职能，注重加强公共资源配置，综合运用政策、规划、标准、技术等手段管理经济，减少政府对微观经济活动的干预。

　　（二）坚持为民执政。牢固树立宗旨意识，切实转变工作作风，帮基层和群众之所需，解基层和群众之所难。大兴调查研究之风，了解基层情况，倾听群众呼声，解决实际问题。坚持听政于民、问政于民，尊重民众的自主权、发挥民众的创造力、提升民众的幸福感

　　（三）坚持依法行政。严格依照法定权限和程序行使权力、履行职责、接受监督，全面落实行政执法责任制和执法过错追究制，加快推进法治政府建设。自觉接受市人大及其常委会的依法监督和市政协的民主监督，认真办理人大代表的议案、建议、批评、意见和政协委员的提案。主动接受民主党派、社会团体、新闻舆论和人民群众监督。深入推进“阳光政务”，创新政务公开方式，扩大公开范围。

　　（四）坚持高效施政。深化行政审批制度改革，精简行政审批事项，优化审批程序。积极推进电子政务、政务网站建设。大力精简会议、文件，减少接待应酬，把主要精力放在上项目、搞建设、干实事、谋发展上，切实增强政府执行力，进一步提高行政效率。

　　（五）坚持廉洁从政。大力倡导艰苦创业、勤俭节约之风，严格控制各类行政经费，做到公务用车、接待、会议、党政机关工作人员出国四项经费支出“零增长”，把有限的资金真正用在促进发展、改善民生上。严格落实党风廉政建设责任制，模范遵守“8个方面禁止”、“52个不准”等廉洁从政规定，强化行政审批、招投标、土地出让、政府采购等重点领域和关键环节监管，严厉查处腐败案件，切实纠正损害群众利益的不正之风。

　　各位代表，站在新的历史起点，适逢新的发展机遇，面对新的压力挑战，让我们在市委的正确领导下，以更加开阔的视野、更加实干的精神、更加昂扬的斗志，坚定信心，迎难而上，为全面开创建设龙江强市、实现更好更快发展的新局面做出更大贡献！
